# Framing the Origins of COVID-19

**DOI:** 10.1177/1075547020953603

**Published:** 2020-10

**Authors:** Toby Bolsen, Risa Palm, Justin T. Kingsland

**Affiliations:** 1 Georgia State University, Atlanta, GA, USA

**Keywords:** framing, COVID-19, conspiracies, public opinion

## Abstract

Conspiracy theories have flourished about the origins of a novel coronavirus (SARS-CoV-2) that causes an acute respiratory syndrome (coronavirus disease 2019 [COVID-19]) in humans. This article reports the results from a study that evaluates the impact of exposure to framed messages about the origins of COVID-19. We tested four hypotheses: two focusing on its origins as either zoonotic or human-engineered and two concerning the impacts of origin beliefs on the desire to penalize China or support increased funding for biomedical research. The results accentuate the importance of finding ways to combat the spread of misinformation and conspiracy theories related to this global pandemic.

## Introduction

Coronavirus disease 2019 (COVID-19) is a novel coronavirus (SARS-CoV-2) that causes an acute respiratory syndrome in humans.^1^ As governments and scientific organizations continue to examine what caused the outbreak, conspiracy theories about its origins have flourished (Ellis, 2020; Van Bavel et al., 2020). It is important to determine the precise origins of the virus and the vectors through which it spreads so that this virus and future similar viruses can be contained, particularly since zoonotic coronaviruses have become an increasing threat to human health, (McMahon et al., 2018; Naicker, 2011; Perlman, 2020; Shereen et al., 2020). From a public health perspective, it is also important to understand any consequences that exposure to conspiracy rhetoric about the origins of COVID-19 might have on the public’s beliefs about the health and public policy implications of the virus as well as their willingness to engage in prosocial behaviors to mitigate its spread.

In this article, we report the results from a survey experiment designed to evaluate the impact of exposure to framed messages about the origins of COVID-19. We focus on two explanations that have received considerable attention: (1) its origins are “zoonotic,” and the virus was transmitted “naturally” from bats to humans, possibly from a food market in Wuhan, China; and (2) a conspiracy theory that it was human-engineered and leaked, deliberately or accidentally, from a research laboratory in Wuhan, China. Social and behavioral scientists have mobilized rapidly to provide crucial insights into the public’s beliefs about COVID-19 (Ballew et al., 2020). We contribute to this line of research by (1) extending research on *emphasis framing effects* to study how scientific information in isolation or in competition with conspiratorial rhetoric affects beliefs about the origins of COVID-19; (2) providing a framework to understand how *belief about the origin* of COVID-19 shapes (a) attributions of responsibility for the pandemic and (b) support for distinct policy responses (e.g., penalize China vs. devote more public funds to researchers studying zoonotic disease transmission); and (3) testing for the presence of a “conspiracy effect” whereby exposure to conspiracy rhetoric reduces individuals’ willingness to engage in prosocial actions (van der Linden, 2015). We find that exposure to framed messages about the origins of COVID-19 can have a powerful impact on beliefs, and in turn, these beliefs about the origin of the virus have powerful “downstream effects” on support for different policies in response to the crisis. In addition, exposure to conspiracy rhetoric in isolation or in competition reduced willingness to engage in prosocial actions to reduce the spread of COVID-19. The results underscore the importance of finding ways to combat scientific misinformation and conspiratorial beliefs that can pose a threat to public health systems (Jerit et al., 2020).

## What We Know About the Origin of COVID-19

News stories about the origins of COVID-19 have proliferated since the onset of the pandemic. Interest in this subject has been driven, in part, by the fact that knowledge about where and how it started is crucial for containing this and similar viruses. Through genetic sequencing, epidemiologists have suggested that the virus started in bats and jumped to humans “naturally,” possibly from people who handled infected animals at a market in Wuhan, China (Ignatius, 2020; Sansonetti, 2020; Sun et al., 2020). Yet the precise animal source of the virus continues to elude scientists, which has led to a proposal by the World Health Organization for “scientific and collaborative field missions” to “identify the zoonotic source of the virus and the route of introduction into the human population, including the possible role of intermediate hosts” (Mallapaty, 2020).

With the uncertainty about the precise origin of the virus, and the tendency of many to want to assess blame, numerous conspiracy theories regarding the origins of the virus have surfaced (Van Bavel et al., 2020). A *conspiracy theory* is an “an effort to explain some event or practice by reference to the machinations of powerful people, who attempt to conceal their role” (Sunstein & Vermeule, 2009, p. 205; see also Lewandowsky, Oberauer, & Gignac, 2013). One theory that has circulated in some conservative journals posits that the virus was accidentally or deliberately leaked from a research laboratory located near the Wuhan market in China where scientists believe the virus originated (Gertz, 2020). Proponents of this conspiracy claim that the virus was deliberately engineered in this laboratory that studies animal coronaviruses to produce an offensive biological weapon. The fact that the Wuhan lab is a branch of the Chinese Center for Disease Control and Prevention and is located about 300 yards from the food market where scientists believe the outbreak started, is pointed out to cast doubt on the “official” conclusion. Despite attempts to “knock down” this unfounded rumor, the idea of the virus as a form of Chinese conspiracy persisted (Abd-Alrazaq et al., 2020; Cinelli et al., 2020; Shahsavari et al., 2020; Uscinski et al., 2020) and has been given support from those, including U.S. President Donald Trump, who persisted in dubbing COVID-19 as the “Chinese virus” (K. Rogers et al., 2020).

## Emphasis Framing and “Origin Beliefs”

Information about the origins of COVID-19, whether “scientific” or “conspiratorial,” is transmitted through *frames in communication* (i.e., “media frames”) that can influence people’s perceptions, beliefs, and actions (Chong & Druckman, 2007b).^2^ An *emphasis framing effect* occurs when exposure to a framed message causes people to prioritize the emphasized consideration(s) when forming a belief (Druckman, 2004).^3^ One recent study, for instance, found that messages that accentuate the public or personal health benefits of practicing COVID-19 prevention behaviors increased respondents’ intentions to engage in these actions (Jordan et al., 2020). More generally, frames in news provide an “interpretive storyline that set(s) a specific train of thought in motion, communicating why an issue might be a problem, who or what might be responsible for it, and what should be done about it” (Nisbet, 2009, p. 15; see also Entman, 1993; Iyengar, 1994).

A voluminous literature demonstrates that exposure to an asymmetric one-sided frame (i.e., exposure to just one argument, see Chong & Druckman, 2007b), such as a statement highlighting the scientific consensus on an issue, can move an audience’s beliefs in the direction of the framed message (Bolsen & Druckman, 2018; Bolsen et al., 2018; Bolsen et al., 2019a, 2019b; Bolsen & Shapiro, 2018; Lewandowsky, Gignac, & Vaughan, 2013; van der Linden et al., 2019). Individuals may learn about the origins of COVID-19 through exposure to stories that communicate either what most scientists believe (i.e., zoonotic transmission) or through exposure to conspiratorial claims (e.g., the virus was created in a research laboratory in China). Druckman (2011) states, “The typical (emphasis) framing effect experiment randomly assigns individuals to receive *one* of two alternative representations of an issue”^4^ (p. 292), and the modal finding in these studies is that individuals to give greater “weight” to the frame that is made salient by the communicator when forming their opinion (Chong & Druckman, 2007b). We extend this line of research to study how presenting people with distinct one-sided arguments about the origin of COVID-19 affects their beliefs when it is encountered in isolation, or as we discuss below, in competitive rhetorical settings. Based on a well-established body of research on how exposure to one-sided frames shape opinion formation, we offer the following prediction:

*Hypothesis 1*: Individuals presented with a one-sided framed message regarding the origin of COVID-19 will shift their belief about its origins in the direction of the frame.

People are often presented with multiple considerations (frames) about any issue within the context of a news story or political debate (Chong & Druckman, 2007b; Sniderman & Theriault, 2004). News coverage surrounding the origins of COVID-19, for instance, may include “competing” narratives within a single story about the virus’s possible origins, such as the consensus scientific position juxtaposed against a prominent conspiracy theory. A “competitive framing” research design refers to an experiment that includes “dual exposure” to distinct (competing) considerations about any issue (Chong & Druckman, 2007b, p. 103).^5^ In such instances, the effect of exposure to “competitive frames” depends on the audience’s perception of the relative “strength” of the competing considerations, as well as other factors such as individual-level motivations that shape information processing (Chong & Druckman, 2007a).

Research on emphasis framing and opinion formation on climate change has demonstrated that exposure to competitive frames that challenge any scientific consensus can undermine its otherwise persuasive impact (Bolsen & Druckman, 2018; van der Linden et al., 2017). Scientific misinformation, “a claim that is unsupported or contradicted by the scientific community’s best available information” (Levy et al., in press, p. 3), can undermine the influence of science on the public and policy makers (Druckman, 2015; Flynn et al., 2017; Van Bavel et al., 2020) and lead to collective decisions that are not in best interests of society (Dietz, 2013; Levy et al., 2020).

The conspiracy narrative that COVID-19 was created in a Wuhan laboratory is an unsubstantiated narrative that challenges the current scientific consensus on the virus’s origins. When individuals are presented with dual (competitive) frames of equal strength, the effects of each message often “cancel out” and result in beliefs similar to those who were not exposed to any information.^6^ We extend the literature on competitive framing by studying how scientific information pitted against a prominent conspiracy theory about the origin of COVID-19 affects individuals’ related perceptions. The application of emphasis framing research to the study of this particular form of scientific misinformation is of urgent importance given the current world stage and the threat COVID-19 presents. Based on prior work that demonstrates that simultaneous exposure to competitive frames cancels out each message’s individual impact, we offer the following prediction:

*Hypothesis 2*: Individuals who are exposed to competitive frames regarding the origins of COVID-19 will express beliefs that are similar to a control condition (that does not receive any information) due to the individual effects of each argument canceling out in competition.

## COVID-19 “Origin Beliefs” and Blame Attributions

Numerous empirical studies have linked the concept of “blame” (the attribution of responsibility for an action or event) with subsequent attitudes and behavior. The concept of blame contains two related but different ideas: cause and responsibility. For example, an event can have a cause, but blame cannot be assigned because the event was unintentional or could not be attributed to a specific actor (Iyengar, 1994; Lagnado & Channon, 2008; Shaver, 1985). Responsibility attribution is central to “everyday reasoning” and is so compelling a concept that people even invent responsibility where none exists (Iyengar, 1994, p. 9). Blame tends to be associated with events that are seen as intentional and also where the outcomes of the action are foreseeable (Alicke, 2000; Ames & Fiske, 2015; R. Rogers et al., 2019).

The attribution of blame to a specific person or group strengthens response: Several empirical studies have demonstrated that when blame is focused on an identifiable target, whether a group or an individual, feelings of anger and a desire for retribution can be elicited, particularly if it can be assumed that the transgressor had free will (I. Levin et al., 2016; Nahmias & Nadelhoffer, 2005; Shariff et al., 2014). Similarly, Javeline (2003) demonstrated that the more specific the attribution of blame, to a particular person, for example, the more people are likely to protest, even if the attribution of blame is inaccurate: “Narrowly attributed blame is a more powerful motivator than diffuse blame, even if diffuse blame is warranted by the objective fact” (p. 108).

Previous research has also demonstrated the effects of frames on the attribution of responsibility (Kim, 2015). For example, Major (2011) investigated the impacts of gain/loss and episodic/thematic frames on perceived responsibility for obesity and lung cancer. He found that specific frames elicited specific emotional responses that, in turn, affected the perceived responsibility as attributed either to society or to the individual.

In addition to the more basic research on framing and blame attribution, there is precedent in recent U.S. history for attributing specific blame to China for a viral epidemic. In 2003, the SARS epidemic caused by another zoonotic transmission of a coronavirus originating in Guandong, China, resulted in widespread fear and blame, fanned by the media in the United States that characterized China and even American-born Chinese as unsanitary and practicing dangerous lifestyles (Eichelberg, 2007). Based on previous findings that the identification of a specific responsible agent increases the desire for retribution, as well as the negative reactions of Americans to China and to Chinese people documented in association with a previous coronavirus outbreak, we hypothesize as follows:

*Hypothesis 3*: Individuals who believe China is responsible for the origin of COVID-19 will be more likely to agree that (a) China should be held financially responsible for the costs associated with the coronavirus outbreak and (b) governments, states, and organizations should be able to sue China to reveal more information about the origin of the coronavirus.

Conversely, respondents who believe that the novel COVID-19 was not created in a Chinese laboratory as a possible bioweapon but instead is one of many zoonotic diseases should have a different perspective. The virus could increase in frequency as a result of two factors: climate change (Bouchard et al., 2019; Fong, 2020; Iwamura et al., 2020; Mills et al., 2010; Naicker, 2011; Ogden & Gachon, 2019; Ogden & Lindsay, 2016) and increased international travel (Chinazzi et al., 2020; Gössling et al., 2020; Wells et al., 2020). Since human vulnerability to the emergence of zoonotic diseases is increasing, these respondents seek remedies to decrease the likelihood of future pandemics. This reasoning motivates the following hypothesis:

*Hypothesis 4*: Individuals who believe COVID-19 had natural origins are more supportive of funding for research to study animal coronaviruses.

## Conspiracy Rhetoric and Prosocial Behaviors

Several studies have tested factors that influence people’s willingness to engage in prosocial actions during the pandemic (Goldberg et al., 2020; Heffner et al., 2020; Jordan et al., 2020; Pennycook et al., 2020). Additionally, in the domain of climate change research, several studies have found that exposure to information stating that it is “hoax” decreases individuals’ willingness to engage in prosocial actions that would reduce their own carbon footprint (Jolley & Douglas, 2014; van der Linden, 2015), a phenomenon referred to as the *conspiracy effect*. We would argue that exposure to conspiracy rhetoric about the origins of COVID-19 might have different effects, because irrespective of what caused the virus, individuals personally benefit from engaging in protective behaviors, in addition to contributing to the provision of a public good.

Despite the recent robust social science research on response to the virus, no one, to our knowledge, has documented how exposure to various origin frames or frames in competition might influence prosocial behavioral intentions. Thus, we pose the following research question:

*Research Question 1*: Does exposure to the Chinese conspiracy origins frame affect individuals’ willingness to engage in voluntary prosocial behaviors (e.g., wearing masks, washing hands, social distancing) to prevent the spread of COVID-19?

## Survey Experiment

We implemented a survey experiment from April 29 to May 3, 2020, in which we randomly assigned 1,071 respondents, recruited from Amazon’s Mechanical Turk (MTurk) to one of four experimental conditions.^7^ Respondents randomly assigned to the *control* condition (*N* = 268) were not exposed to any information prior to answering our key outcome measures (described below). Respondents randomly assigned to one of the other three conditions were exposed to a message that varied the headline and content of a short article formatted to mimic a news story about the origin of COVID-19 (Table 1). We restricted the sample to U.S. respondents who had successfully completed at least 100 tasks and had at least a 95% approval rating on MTurk. The median completion time for the survey was 5.8 minutes, and participants received $0.25 in remuneration on completion.^8^ The sample was large and diverse with respect to demographic and political characteristics: For instance, 33% of respondents identified as Republicans, 27% identified as Independents, and 40% identified as Democrats. Furthermore, our sample is 45% female and 55% male. Other descriptive statistics for the sample are available online in the Supplemental Appendix.

**Table 1.** Experimental Treatments.

Condition	Message
Control (*N* = 268)	Please answer the following questions.
Natural origin (*N* = 270)	*Coronavirus originated in animals and jumped to humans* Many infectious diseases are “zoonotic.” This means they start in animals but jump to humans. In fact, 6 out of every 10 infectious diseases in people are zoonotic. This is true of the COVID-19 virus that genetic sequencing has shown originated in bats and was naturally transmitted to humans. Coronaviruses are part of a large family of viruses that are common in people and many different species of animals, including camels, cattle, cats, and bats. Animal coronaviruses have infected people in the past and will continue to be a threat. The disease known as “mad cow disease” came from people eating cattle, and just a few weeks ago a fatal strain of bird (avian) flu was detected in a commercial turkey flock in the United States. Many of the patients at the epicenter of the coronavirus outbreak in Wuhan, China, had some link to a large seafood and live animal market, indicating animal-to-person origins. Such marketplaces have been breeding grounds for animal viruses that run rampant and get passed on to humans. Scientists believe this was what caused the current outbreak.
Chinese conspiracy (*N* = 266)	*Coronavirus originated in a Chinese laboratory* Many U.S. intelligence officials and scientists believe that the coronavirus originated from an accidental leak at a research laboratory located in Wuhan, China. The Wuhan Institute of Virology is a research laboratory connected with the Chinese Center for Disease Control and Prevention, which is located about 300 yards from the marketplace where some claim the virus started. The Wuhan lab produces research on offensive biological warfare weapons, and the creation of synthetic viruses linked to animals. Many believe that either an accidental or deliberate leak of the virus from the Wuhan lab is what caused the current outbreak. While genetic sequencing has been used to show that COVID-19 exists in bats, there are no bats sold at the seafood market in Wuhan. In addition, a study published in the Lancet found that the first coronavirus case in China had no connection to the Wuhan market. Instead, improper safety measures at the Wuhan laboratory likely led to an accidental outbreak of a human-created virus.
Competitive frame (*N* = 267)	*Did the coronavirus originate in a Chinese laboratory or naturally in animals?* There has been a debate among scientists and other about the origin of the novel coronavirus disease (COVID-19). Some have said that like many other “zoonotic” or animal-based viruses, it originated in an animal species and jumped to humans, from which it spread from person to person. Coronaviruses are part of a large family of viruses that are common in people and many different species of animals, including camels, cattle, cats, and bats. Animal coronaviruses have infected people in the past and will continue to be a threat. Others argue that the coronavirus originated from an accidental leak at a research laboratory located in Wuhan, China. The Wuhan Institute of Virology is a research laboratory connected with the Chinese Center for Disease Control and Prevention, which is located about 300 yards from the marketplace where some claim the virus started. A study published in the Lancet found that the first coronavirus case in China had no connection to the Wuhan market. Instead, improper safety measures at the Wuhan laboratory likely led to an accidental outbreak of a human-created virus.

Participants in all conditions were informed at the beginning of the survey that they would be asked some questions about their opinions related to COVID-19. Respondents in the control condition immediately proceeded to answer the key outcome measures. All other participants were exposed to one of the experimental treatments immediately before responding to the dependent measures. Respondents randomly assigned to the *natural origin* condition (*N* = 270) were presented with the headline, “Coronavirus Originated in Animals and Jumped to Humans,” followed by details in a short article that included statements such as “Many infectious diseases are “zoonotic” . . . [which means] they start in animals but jump to humans,” “This is true of the COVID-19 virus which genetic sequencing has shown originated in bats and was naturally transmitted to humans,” and “Scientists believe this was what caused the current outbreak.” The complete wording of the experimental treatment for all conditions is reported in Table 1.

Respondents randomly assigned to the *Chinese conspiracy* condition (*N* = 266) were presented with the headline, “Coronavirus Originated in a Chinese Laboratory,” followed by details in a short article that included statements such as “the coronavirus [may have] originated from [a] leak at a research laboratory located in Wuhan, China.” It further explained that the Wuhan Institute of Virology’s research laboratory is located about 300 yards from the market where some claim the virus started” [and that]

the Wuhan lab produces research on offensive biological warfare weapons and the creation of viruses linked to animals. Many believe that either an accidental or deliberate leak of the virus from the Wuhan lab is what caused the current outbreak.

It also stated that “while genetic sequencing has been used to show that COVID-19 exists in bats, there are no bats sold at the seafood market in Wuhan.”

Respondents assigned to the *competitive framing* condition (*N* = 267) were presented with the headline, “Did the Coronavirus Originate in a Chinese Laboratory or Naturally in Animals?” followed by details in a short article that included information selected from both the natural origin and the Chinese conspiracy conditions. It stated, “There has been a debate among scientists and other about the origins of the novel coronavirus (COVID-19).”

To complete the survey, respondents had to check a box to indicate they had read the following debriefing statement:

Although there is controversy in some quarters about the ultimate cause of the virus that causes COVID-19, there is absolutely no evidence at all that the virus was engineered as part of a weapons program. For factual information about the source, symptoms and mitigation measures, please consult the website from the Centers for Disease Control and Prevention. Link: (https://www.cdc.gov/coronavirus/2019-ncov/index.html)

## Dependent Measures

Participants, except for those in the control condition, read a version of the “short article” and then reported the extent to which they believed “the coronavirus originated in animals and jumped to humans versus originating in a laboratory in China?” on a 7-point scale (1 = *definitely originated in animals*; 7 = *definitely created in a laboratory*). We also asked respondents how likely they believe it is that the “coronavirus originated in animals and jumped to humans” on a 7-point scale (1 = *extremely unlikely*; 7 = *extremely likely*), how likely it is that the “coronavirus originated in a laboratory in China” (1 = *extremely unlikely*; 7 = *extremely likely*), and the extent to which respondents agreed with the statement “The coronavirus was created by the Chinese government as part of a biological weapons program” (1 = *strongly disagree*; 7 = *strongly agree*). These four items formed a reliable index (α *=* .84), which we used to create our measure *origin beliefs*, and coded so that higher scores are associated with a greater belief that the virus was created in a Chinese laboratory.

Second, we measured the extent to which respondents disagreed or agreed with the statements, (a) “China should be held financially responsible for the costs associated with the outbreak” and (b) “Governments, states, and organizations should be able to sue China to reveal more information about the origin and spread of the coronavirus” (1 = *strongly disagree*; 7 = *strongly agree*). These two items formed a reliable index (α *=* .88), and constitute our measure *penalize China*.

Third, we measured the extent to which respondents opposed or supported “the U.S. government increasing spending for research on zoonotic (animal-transmitted) coronaviruses” (1 = *strongly oppose*; 7 = *strongly support*), which we refer to as support for *biomedical research*. Fourth, we asked respondents how necessary it has been to (a) wear facemasks, (b) frequently wash hands, and (c) maintain six feet of distance in social settings on a 7-point scale (1 = *not necessary at all*; 7 = *extremely necessary*). These three items formed a reliable index (α *=* .95), which we labeled *prosocial behavior* and coded so that higher scores indicate greater perceived necessity of engaging in these actions.

## Results

To test our hypotheses, we estimate a collection of ordinary least squares regression models with robust standard errors. We regress each dependent variable on our condition indicators, omitting the *Control* condition as the reference group. The results of our analysis are reported in Tables 2 and 3;^9^ additional analyses using alternative model specifications are included in the Supplemental Appendix (available online).^10^

**Table 2.** Main Effects.

Variable	Origin beliefs			Penalize China			Biomedical research		
	Coefficient	*p*	95% CI	Coefficient	*p*	95% CI	Coefficient	*p*	95% CI
Natural origin	−0.33** (0.13)	.011	[−0.59, −0.08]	0.08 (0.13)	.525	[−0.17, 0.34]	0.10 (0.12)	.420	[−0.14, 0.33]
Chinese conspiracy	0.40*** (0.13)	.002	[0.14, 0.65]	−0.01 (0.13)	.936	[−0.27, 0.25]	−0.11 (0.12)	.339	[−0.35, 0.12]
Competitive frame	0.24* (0.13)	.069	[−0.02, 0.49]	−0.25* (0.13)	.055	[−0.50, 0.01]	0.11 (0.12)	.373	[−0.13, 0.34]
Origin beliefs				0.63*** (0.03)	.000	0.57, 0.69	−0.22*** (0.03)	.000	[−0.28, −0.17]
Constant (control)	3.44*** (0.09)	0.000	3.26, 3.63	2.28*** (0.14)	0.000	2.00, 2.55	5.98*** (0.13)	.000	5.73, 6.23
*N*	1,071			1,071			1,071		
AIC	3915.3			3911.2			3716.1		
BIC	3935.2			3936.1			3740.9		

*Note.* Cell entries are ordinary least squares coefficients with standard errors in parentheses. Two-tailed *p* values and confidence intervals are presented in the adjacent columns. Coefficient estimates for the condition indicators represent the difference in means between the treatment condition and the control group baseline. The coefficient for origin beliefs represents the estimated effect of a one-unit increase on the origin beliefs scale on the outcome measures in models for penalize China and biomedical research. AIC = Akaike information criterion; BIC = Bayesian information criterion; CI = confidence interval.

* *p* < .10. ^**^p < .05. ^***^p < .01.

**Table 3.** Prosocial Action.

Variable	Coefficient	*p*	95% CI
Natural origin	−0.16 (0.10)	.121	[−0.36, 0.04]
Chinese conspiracy	−0.26** (0.10)	.011	[−0.46, −0.06]
Competitive frame	−0.21** (0.10)	.041	[−0.41, −0.01]
Constant (control)	6.10*** (0.07)	.000	[5.96, 6.25]
*N*	1,071		
AIC	3408.8		
BIC	3428.7		

*Note.* Cell entries are ordinary least squares coefficients with standard errors in parentheses. Two-tailed *p* values and confidence intervals are presented in the adjacent column. Coefficient estimates represent the difference in means between the treatment condition and the control group baseline. AIC = Akaike information criterion; BIC = Bayesian information criterion; CI = confidence interval.

* *p* < .10. ^**^p < .05. ^***^p < .01.

### Origin Beliefs

Our first hypothesis was that one-sided frames would influence beliefs about the origin of COVID-19. As we predicted (Hypothesis 1), respondents who read the *natural origin* treatment were more likely to believe that the virus started in animals and jumped naturally to humans versus being created in a research laboratory in China (*b =* −0.33, *p =* .01, left-hand column, Table 2).^11^ Similarly, respondents who read the *Chinese conspiracy* treatment were more likely to believe that the virus was created in a Chinese laboratory as opposed to an accidental animal to human transmission (*b =* 0.40, *p =* .01).

Our second hypothesis was that competing frames would reduce the impact of the one-sided frames and that the opinions of those exposed to competing frames about the origin of the virus differ from the control group baseline. Counter to our prediction (Hypothesis 2), the results show no statistically significant effect for the competitive framing condition: Respondents who read the *competitive framing* story about the virus’s origin did not significantly differ from the control group with regard to the belief that COVID-19 was created in a research laboratory in China (*b =* 0.24, *p =* .07), although this direction did not reach the threshold of statistical significance.

### Willingness to Penalize China

The experimental treatments we designed emphasizing different narratives about the origin of COVID-19 had a powerful impact on people’s beliefs about the origin of the virus. We theorized that people’s *origin beliefs* are important because they may have downstream impacts on opinions about the appropriate policy responses from governments to address this as well as future pandemics. The middle column of results in Table 2 shows the indirect effect of the experimental treatments—through their impact on “origin beliefs”—on support for efforts to hold China financially responsible for the COVID-19 outbreak.^12^ As we predicted (Hypothesis 3), respondents who believe the coronavirus originated in a Chinese laboratory, as opposed to zoonotic transmission, are more willing to penalize China for the outbreak through policies that target financial restitution (*b =* 0.63, *p* < .01). The effect of origin beliefs is substantively large, as each one-unit increase corresponds with an expected increase of 0.63 on the outcome measure (*p <* .01).

### Support for Biomedical Research

The right-hand column of results in Table 2 illustrates the indirect effect of the treatments—through their impact on “origin beliefs”—on support for additional funding for biomedical research to identity the nature of zoonotic coronaviruses that pose a threat to humans. In support of our prediction (Hypothesis 4), respondents who believe the virus originated naturally in animals and was transmitted to humans were more supportive of additional research funds for scientists to study zoonotic viruses; each one-unit increase in the belief that the virus has *unnatural* origins corresponds with a 0.22 *decrease* in support for research funding (*b = −*0.22, *p =* .000).

### Prosocial Behavior

We also evaluate the effect that exposure to a conspiracy theory about the origins of COVID-19 exerted on respondents’ willingness to engage in actions (*prosocial behavior*) such as wearing facemasks, frequently washing hands, and maintaining at least 6 feet of distance from other people outside the home as necessary for preventing its spread (Research Question 1). Table 3 reports clear evidence for the effect of a “conspiracy effect”: Exposure to the conspiracy frame in isolation (*b =* –0.26, *p =* .01) and in the competitive framing condition (*b =* –0.21, *p =* .04) reduced the perceived necessity of engaging in these prosocial behaviors.

## Conclusion

At this time, scientists remain uncertain about the precise origins of COVID-19. In an atmosphere of uncertainty and fear, conspiracy theories about COVID-19’s origins have spread. Van Bavel et al. (2020) explain the linkage between fear and conspiracy in this way: “People feel the need to explain large events with proportionally large causes and are more likely to believe in conspiracy theories about events with serious consequences and in times of crisis” (p. 464; see also Van Prooijen & Douglas, 2017). Public acceptance of conspiracy narratives, however, can be harmful not only because it can lead people to dismiss credible science but also because it can reduce the perceived importance of engaging in behaviors that are individually and collectively beneficial (Lewandowsky, Oberauer, & Gignac, 2013; Oliver & Wood, 2014; Uscinski et al., 2017), including potentially life-saving actions such as following the recommendations of public health experts to mitigate COVID-19’s spread (Jerit et al., 2020).

We find that exposure to framed messages regarding the origins of COVID-19 can have a powerful effect on people’s beliefs about the cause of this global pandemic. Moreover, beliefs about the origin of the virus had strong “downstream effects” on respondents’ willingness to penalize China when they believe it may have been created by the Chinese government. Conversely, those who believe the virus originated naturally from zoonotic transmission were more supportive of additional funding for biomedical research to identify harmful coronaviruses. Finally, exposure to a conspiracy theory about the virus’s origin, in isolation or in competition, resulted in a “conspiracy effect” whereby individuals became less likely to view actions such as wearing facemasks, frequently washing one’s hands, and maintaining 6 feet of social distance as necessary in order to mitigate COVID-19’s spread.

This demonstration of the conspiracy effect in this domain is novel and important; however, it is also potentially worrisome insofar as a single exposure to a conspiracy theory in our study reduced individuals’ intentions to practice urgently necessary public health behaviors. Furthermore, the contemporary media environment is competitive and people tend to consume media that fits and reinforces their existing perspectives. In this environment, some individuals may be exposed to conspiracy messages repeatedly. As a result, our findings may actually understate the effects of exposure to conspiracy rhetoric—especially given that we used textual frames as treatments to challenge the scientific frames as opposed to videos or other visual frames that can be even more impactful on audiences (Goldberg et al., 2019; van der Linden, 2015).

It is crucial for future research to extend the findings we report in several ways. First, as with any study, it is important to consider how aspects such as the timing (i.e., when the study was conducted) and decisions regarding the content of the experimental treatments may have influenced our outcomes, as well as specific individual-level factors that may moderate the effects of exposure to the frames we employed. The decision to use relatively short textual “news articles,” in isolation and competition, was undertaken to shed light on how scientific and conspiratorial frames affect people’s beliefs and actions surrounding the origin of COVID-19, shape perceptions of who is responsible, and influence personal behaviors. Future work on larger, representative samples is needed to determine how individual-level factors—such as party identification, values/worldviews, general conspiratorial beliefs or other factors—may moderate the main treatment effects we reported.

Second, given the powerful effects of a single exposure to our experimental treatments, it is important for future research to account for how repeated exposure to specific conspiracy theories may influence related beliefs in settings that more accurately mimic real-world information environments. This would require longitudinal experimental designs that vary the frequency of a particular conspiracy theory; however, it would also provide an opportunity to assess the duration, or persistence, of the effects of scientific and conspiratorial frames on audiences.

Third, it is crucial find ways to combat the powerful effects that exposure to scientific misinformation, such as in the form of conspiracy theories and fake news, can exert on audiences. Our research shows that in a competitive rhetorical setting surrounding debate over the origins of COVID-19, conspiracy rhetoric can have a profound impact and overpower scientific information. However, our study was not designed to test for ways to combat the effects of exposure to the conspiracy rhetoric, for instance, by including additional conditions that provide a warning that one will be exposed to inaccurate or misleading information (i.e., inoculation) or through “corrective” information that debunks a conspiracy theory following exposure to it.

The spread of the COVID-19 virus has been an accompanying epidemic of misinformation, eroding trust in science and misleading individuals about the most effective precautions to take to quell the virus and ensure safety. It is urgent that as we seek to control the spread of this virus and anticipate ways to control and suppress future similar viruses, we come up with ways to combat misleading and damaging conspiracy rhetoric.

## Supplemental Material

Supplementary_Appendix_8.6.20 – Supplemental material for Framing the Origins of COVID-19 Click here for additional data file. Supplemental material, Supplementary_Appendix_8.6.20 for Framing the Origins of COVID-19 by Toby Bolsen, Risa Palm and Justin T. Kingsland in Science Communication

## Notes

1. The website of the Centers for Disease Control and Prevention in the United States notes that at this point “the exact source of this virus is unknown” although the virus is similar to MERS-CoV and SARS-CoV that had its origin in animal reservoirs (https://www.cdc.gov/coronavirus/2019-ncov/faq.html, accessed June 16, 2020).

2. A *frame* refers to words, phrases, or symbols that highlight a subset of the potentially relevant evaluative dimensions associated with any attitude object (Druckman, 2001).

3. We focus exclusively on emphasis framing effects and not equivalency or valence framing effects that occur when positive or negative information unconsciously influences preferences as a result of a negativity bias in the encoding of stimulus information (Tversky & Kahneman, 1981; for a typology of framing effects, see I. P. Levin et al., 1998).

4. There are instances where frame exposure will not influence people’s opinions; for instance, individuals may sometimes possess strong prior beliefs or values that “prevent a frame from exerting an effect” (Druckman, 2011, p. 283). There is a substantial body of research on the moderators of exposure to one-sided frames in communication (see Chong & Druckman, 2007b, pp. 111-112; Druckman & Leeper, 2012).

5. Chong and Druckman (2007b) state, “Asymmetric one-sided studies are therefore “noncompetitive” because individuals are exposed to only one side of a controversy, whereas [dual two-sided] designs model ‘competitive’ environments” (p. 103).

6. However, when scientific misinformation is encountered prior to establishing a belief, it can be difficult to correct misperceptions due to the persistent impact of exposure to unsubstantiated information (Thorson, 2016; Walter & Tukachinsky, 2020). The study reported here did not attempt to examine either belief persistence or inoculation, but instead focuses on competitive framing of specific messages.

7. MTurk is a widely used online crowdsourcing platform that generates more diverse samples than many randomized experiments that rely on student participants (Berinsky et al., 2012). As with other convenience samples, MTurk differs in several ways from a general population sample (but not in ways that impede making generalizable causal inferences, see Levay et al., 2016); for instance, participants tend to be more educated and express higher levels of political interest. Nonetheless, it is commonly used in the social sciences to estimate causal relationships, and the results are comparable to identical studies fielded on general population samples (Mullinix et al., 2015).

8. We did not have a predefined sample size prior to fielding the study, but instead sought to maximize the number of participants recruited given total cost considerations and the research budget.

9. As an additional test, we reestimated our empirical models with several demographic and political covariates included. The results are reported in Supplemental Tables A2 and A3 (available online). We do not report these models in our main analysis for two reasons. First, we conducted randomized experiment, and given the randomization procedure was successfully implemented, the inclusion of individual level covariates should not change the substantive conclusions derived from results we report. And second, our theoretical framework and hypotheses are primarily concerned with examining the effects of the experimental manipulations on our dependent measures. However, as reported in Supplemental Tables A2 and A3, our results are robust to alternative specification, and generally, show improved precision (e.g., a reduction in error and associated *p* values).

10. An additional analysis probing the impact of party identification and a test for interaction effects is reported in Supplemental Tables A4 and A5 (available online). In these models, we include the condition indicators, a 7-point measure (ranging from 1 = *strong Republican* to 7 = *strong Democrat*) of respondents’ party identification, and interaction terms for party identification and each condition indicator. In each of the additional models, the estimates show a statistically significant relationship between party identification and mean responses to questions about theories of the origin of the virus and to issues such as the willingness to penalize China or adopt particular public health measures. What we did not find, however, was any partisan difference *in the effect of our treatments*: There was no statistically significant interaction between party identification (or ideology) and the relationship between the treatments and responses.

11. The coefficient estimates for origin beliefs represent the estimated effect on the dependent variable resulting from a one-unit change in the origin beliefs scale.

12. Our analysis follows the causal steps approach proposed by Baron and Kenny (1986) for the simple mediation model. We estimate sequential models; first, we regress our measure of origin beliefs on our condition indicators, and then we regress the dependent measure on the condition indicators and our measure of origin beliefs.
