# Supplementary material for: Framing the Origins of COVID-19
Source: Sci Commun. 2020 Oct;42(5):562–85. doi: 10.1177/1075547020953603 (PMC7484600; doi:10.1177/1075547020953603)
Supplement: Supplementary_Appendix_8.6.20 – Supplemental material for Framing the Origins of COVID-19 [file Supplementary_Appendix_8.6.20.docx]

# **Supplementary Appendix: Table A1: Descriptive Statistics and Demographics**

| \| **Variable** \| Total: (N = 1071) \| \| --- \| --- \| |
| --- | --- | --- |
| \| **Origin Belief Scale** \| Mean (SD): 3.52 (1.53) \| \| --- \| --- \| \| **Cost & Sue** \| Mean (SD): 4.46 (1.78) \| \| **Research** \| Mean (SD): 5.23 (1.41) \| \| **Personal Steps Scale** \| Mean (SD): 5.95 (1.19) \| \| **Variable** \| **Frequency (%)** \| \| **Republican** \| 355 (33.1%) \| \| **Independent** \| 284 (26.5%) \| \| **Democrat** \| 432 (40.3%) \| \| **Age** \|  \| \| 18 - 24 \| 121 (11.3%) \| \| 25 - 34 \| 370 (34.5%) \| \| 35 - 44 \| 263 (24.6%) \| \| 45 - 54 \| 160 (14.9%) \| \| 55 - 64 \| 94 (8.8%) \| \| 65 - 74 \| 57 (5.3%) \| \| 75 - 84 \| 5 (0.5%) \| \| 85 or older \| 1 (0.1%) \| \| **Female** \|  \| \| Male \| 476 (44.8%) \| \| Female \| 586 (55.2%) \| \| **Race** \|  \| \| White \| 784 (73.2%) \| \| African American \| 103 (9.6%) \| \| Asian American \| 102 (9.5%) \| \| Hispanic \| 62 (5.8%) \| \| Other \| 20 (1.9%) \| \| **Education** \|  \| \| Less than high school \| 3 (0.3%) \| \| High school graduate \| 86 (8.0%) \| \| Some college \| 203 (19.0%) \| \| 2 year degree \| 85 (7.9%) \| \| 4 year degree \| 478 (44.6%) \| \| Professional degree \| 188 (17.6%) \| \| Doctorate \| 28 (2.6%) \| \| **Income** \|  \| \| Less than $10,000 \| 39 (3.6%) \| \| $10,000 - $19,999 \| 64 (6.0%) \| \| $20,000 - $29,999 \| 101 (9.4%) \| \| $30,000 - $39,999 \| 117 (10.9%) \| \| $40,000 - $49,999 \| 122 (11.4%) \| \| $50,000 - $59,999 \| 123 (11.5%) \| \| $60,000 - $69,999 \| 96 (9.0%) \| \| $70,000 - $79,999 \| 84 (7.8%) \| \| $80,000 - $89,999 \| 48 (4.5%) \| \| $90,000 - $99,999 \| 65 (6.1%) \| \| $100,000 - $149,999 \| 151 (14.1%) \| \| More than $150,000 \| 61 (5.7%) \| |

**Appendix: Dependent Measures**

*Dependent Measures*

To what extent do you believe the coronavirus originated in animals and jumped to humans versus originating in a laboratory in China?

1. Definitely originated in animals (1)
2. Very likely originated in animals (2)
3. Probably originated in animals (3)
4. Not sure (4)
5. Probably created in a laboratory (5)
6. Very likely created in a laboratory (6)
7. Definitely created in a laboratory (7)

How likely is it to you that the coronavirus originated in animals and jumped to humans?

1. Extremely unlikely (1)
2. Moderately unlikely (2)
3. Slightly unlikely (3)
4. Neither likely nor unlikely (4)
5. Slightly likely (5)
6. Moderately likely (6)

Extremely likely (7)

(Reverse coded)

How likely is it to you that the coronavirus originated in a laboratory in China?

1. Extremely unlikely (1)
2. Moderately unlikely (2)
3. Slightly unlikely (3)
4. Neither likely nor unlikely (4)
5. Slightly likely (5)
6. Moderately likely (6)

Extremely likely (7)

To what extent do you disagree or agree with the following statement:

The coronavirus was created by the Chinese government as part of a biological weapons program.

1. Strongly disagree (1)
2. Disagree (2)
3. Somewhat disagree (3)
4. Neither agree nor disagree (4)
5. Somewhat agree (5)
6. Agree (6)

Strongly agree (7)

How necessary has it been to take the following steps related to the coronavirus:

- - - - 1. Wearing Face Masks
        2. Frequently Washing Hands
        3. Maintaining 6 feet of distance in social settings

Not at all necessary (1)

Very unnecessary

Somewhat unnecessary

Neither unnecessary nor necessary

Somewhat necessary

Very necessary

Extremely necessary (7)

*Demographic Measures*

Age - How old are you?

Under 18 (1)

18 - 24 (2)

25 - 34 (3)

35 - 44 (4)

45 - 54 (5)

55 - 64 (6)

65 - 74 (7)

75 - 84 (8)

85 or older (9)

Female - What is your sex?

Male (0)

Female (1)

Education - What is the highest level of education you have completed?

Less than high school (1)

High school graduate (2)

Some college (3)

2 year degree (4)

4 year degree (5)

Professional degree (6)

Doctorate (7)

Income - What is your estimate of your family’s annual household income (before taxes)?

Less than $10,000 (1)

$10,000 - $19,999 (2)

$20,000 - $29,999 (3)

$30,000 - $39,999 (4)

$40,000 - $49,999 (5)

$50,000 - $59,999 (6)

$60,000 - $69,999 (7)

$70,000 - $79,999 (8)

$80,000 - $89,999 (9)

$90,000 - $99,999 (10)

$100,000 - $149,999 (11)

More than $150,000 (12)

Party Identification - Generally speaking, which of the options on the scale below best describes your party identification?

Strong Republican (1)

Weak Republican (2)

Lean Republican (3)

Independent (4)

Lean Democrat (5)

Weak Democrat (6)

Strong Democrat (7)

Ideology - Which point on this scale best describes your political views?

Very conservative (1)

Mostly conservative (2)

Somewhat conservative (3)

Moderate (4)

Somewhat liberal (5)

Mostly liberal (6)

Very liberal (7)

**Appendix: Alternative Model Specifications with Demographics**

***Table A2 – Main Effects with Demographics***

|  |  | Origin Beliefs | | |  | Penalize China | | |  | Biomedical Research | | |  |
| --- | --- | --- | --- | --- | --- | --- | --- | --- | --- | --- | --- | --- | --- |
|  |  |  |  |  |  |  |  |  |  |  |  |  |  |
|  |  | *Coef.* | *p-value* | *95% CI* |  | *Coef.* | *p-value* | *95% CI* |  | *Coef.* | *p-value* | *95% CI* |  |
| Natural Origin |  | -0.24** | 0.043 | -0.48, -0.01 |  | 0.06 | 0.615 | -0.18, 0.31 |  | 0.10 | 0.381 | -0.13, 0.33 |  |
|  |  | (0.12) |  |  |  | (0.13) |  |  |  | (0.12) |  |  |  |
| Chinese Conspiracy |  | 0.40*** | 0.001 | 0.16, 0.64 |  | -0.03 | 0.836 | -0.28,0.22 |  | -0.12 | 0.322 | -0.35, 0.11 |  |
|  |  | (0.12) |  |  |  | (0.13) |  |  |  | (0.12) |  |  |  |
| Competitive Frame |  | 0.26** | 0.033 | 0.02, 0.49 |  | -0.26** | 0.037 | -0.51, -0.02 |  | 0.09 | 0.419 | -0.14, 0.32 |  |
|  |  | (0.12) |  |  |  | (0.13) |  |  |  | (0.12) |  |  |  |
| Party Identification |  | -0.11*** | 0.001 | -0.18, -0.05 |  | -0.06* | 0.073 | -0.13, 0.01 |  | 0.04 | 0.191 | -0.02, 0.10 |  |
|  |  | (0.03) |  |  |  | (0.03) |  |  |  | (0.03) |  |  |  |
| Ideology |  | -0.21*** | 0.000 | -0.28, -0.14 |  | -0.13*** | 0.001 | -0.20, -0.05 |  | 0.08** | 0.024 | 0.01, 0.15 |  |
|  |  | (0.04) |  |  |  | (0.04) |  |  |  | (0.04) |  |  |  |
| Age |  | -0.03 | 0.404 | -0.09, 0.04 |  | -0.04 | 0.191 | -0.11, 0.02 |  | 0.12*** | 0.000 | 0.06, 0.18 |  |
|  |  | (0.03) |  |  |  | (0.03) |  |  |  | (0.03) |  |  |  |
| Income |  | -0.05*** | 0.001 | -0.08, -0.02 |  | -0.01 | 0.594 | -0.04, 0.02 |  | 0.03** | 0.011 | 0.01, 0.06 |  |
|  |  | (0.01) |  |  |  | (0.01) |  |  |  | (0.01) |  |  |  |
| Education |  | -0.06* | 0.089 | -0.13, 0.01 |  | -0.07* | 0.053 | -0.14, 0.00 |  | -0.06* | 0.096 | -0.12, 0.01 |  |
|  |  | (0.03) |  |  |  | (0.04) |  |  |  | (0.03) |  |  |  |
| Female |  | 0.27*** | 0.002 | 0.10, 0.44 |  | -0.32*** | 0.000 | -0.50, -0.14 |  | -0.01 | 0.918 | -0.17, 0.16 |  |
|  |  | (0.09) |  |  |  | (0.09) |  |  |  | (0.08) |  |  |  |
| Origin Beliefs |  |  |  |  |  | 0.56*** | 0.000 | 0.50, 0.63 |  | -0.17*** | 0.000 | -0.22, -0.11 |  |
|  |  |  |  |  |  | (0.03) |  |  |  | (0.03) |  |  |  |
| Constant (Control) |  | 5.30*** | 0.000 | 4.80, 5.79 |  | 4.04*** | 0.000 | 3.42, 4.65 |  | 4.83*** | 0.000 | 4.26, 5.41 |  |
|  |  | (0.25) |  |  |  | (0.31) |  |  |  | (0.29) |  |  |  |
| N |  | 1062 |  |  |  | 1062 |  |  |  | 1062 |  |  |  |
| *AIC* |  | 3717.1 |  |  |  | 3809.3 |  |  |  | 3651.0 |  |  |  |
| *BIC* |  | 3766.8 |  |  |  | 3864.0 |  |  |  | 3705.6 |  |  |  |

*Note:* Cell entries are OLS coefficients with standard errors in parentheses below. Two-tailed *p*-values and confidence intervals are presented in the adjacent columns. Coefficient estimates for the condition indicators represent the difference in means between the treatment condition and the Control group baseline. The coefficient for *Origin Beliefs* represents the estimated effect of a one-unit increase on the *Origin Beliefs* scale on the outcome measures in models for *Penalize China* and *Biomedical Research.*

** p<0.10, ** p<0.05, *** p<0.01*

**Appendix: Alternative Model Specifications with Demographics**

***Table A3 - Personal Steps with Demographics***

|  | *Coef.* | *p-value* | *95% CI* |
| --- | --- | --- | --- |
| Natural Origin | -0.17* | 0.075 | -0.36, 0.02 |
|  | (0.10) |  |  |
| Chinese Conspiracy | -0.24** | 0.014 | -0.43, -0.05 |
|  | (0.10) |  |  |
| Competitive Frame | -0.20** | 0.037 | -0.39, -0.01 |
|  | (0.10) |  |  |
| Party Identification | 0.05* | 0.061 | -0.00, 0.10 |
|  | (0.03) |  |  |
| Ideology | 0.14*** | 0.000 | 0.08, 0.20 |
|  | (0.03) |  |  |
| Age | 0.07*** | 0.009 | 0.02, 0.12 |
|  | (0.03) |  |  |
| Income | 0.04*** | 0.000 | 0.02, 0.06 |
|  | (0.01) |  |  |
| Education | -0.06** | 0.027 | -0.12, -0.01 |
|  | (0.03) |  |  |
| Female | 0.28*** | 0.000 | 0.15, 0.42 |
|  | (0.07) |  |  |
| Constant (Control) | 4.90*** | 0.000 | 4.50, 5.30 |
|  | (0.20) |  |  |
| N | 1062 |  |  |
| *AIC* | 3262.6 |  |  |
| *BIC* | 3312.2 |  |  |

*Note:* Cell entries are OLS coefficients with standard errors in parentheses below. Two-tailed *p*-values and confidence intervals are presented in the adjacent columns. Coefficient estimates for the condition indicators represent the difference in means between the treatment condition and the Control group.

** p<0.10, ** p<0.05, *** p<0.01*

**Appendix: Alternative Model Specification – Treatment Effects and Partisanship**

***Table A4: Condition Assignment and Party Identification***

|  | Origin Beliefs | | |  | Penalize China | | |  | Biomedical Research | | |  |
| --- | --- | --- | --- | --- | --- | --- | --- | --- | --- | --- | --- | --- |
|  | *Coef.* | *p-value* | *95% CI* |  | *Coef.* | *p-value* | *95% CI* |  | *Coef.* | *p-value* | *95% CI* |  |
| Natural Origin | -0.46 | 0.117 | -1.03, 0.11 |  | 0.28 | 0.359 | -0.31, 0.87 |  | 0.17 | 0.549 | -0.38, 0.71 |  |
|  | (0.29) |  |  |  | (0.30) |  |  |  | (0.28) |  |  |  |
| Chinese Conspiracy | 0.22 | 0.446 | -0.34, 0.77 |  | 0.21 | 0.478 | -0.37, 0.78 |  | 0.09 | 0.735 | -0.44, 0.62 |  |
|  | (0.28) |  |  |  | (0.29) |  |  |  | (0.27) |  |  |  |
| Competitive Frame | 0.37 | 0.184 | -0.18, 0.92 |  | -0.28 | 0.328 | -0.85, 0.28 |  | 0.07 | 0.799 | -0.46, 0.59 |  |
|  | (0.28) |  |  |  | (0.29) |  |  |  | (0.27) |  |  |  |
| Party Identification | -0.26*** | 0.000 | -0.34, -0.17 |  | -0.14*** | 0.003 | -0.23, -0.05 |  | 0.09** | 0.031 | 0.01, 0.17 |  |
|  | (0.04) |  |  |  | (0.05) |  |  |  | (0.04) |  |  |  |
| Natural Origin X Party Identification | 0.05 | 0.446 | -0.07, 0.17 |  | -0.04 | 0.545 | -0.17, 0.09 |  | -0.02 | 0.731 | -0.14, 0.10 |  |
|  | (0.06) |  |  |  | (0.06) |  |  |  | (0.06) |  |  |  |
| Chinese Conspiracy X Party Identification | 0.05 | 0.462 | -0.08, 0.17 |  | -0.05 | 0.478 | -0.17, 0.08 |  | -0.05 | 0.366 | -0.17, 0.06 |  |
|  | (0.06) |  |  |  | (0.06) |  |  |  | (0.06) |  |  |  |
| Competitive Frame X Party Identification | -0.02 | 0.707 | -0.14, 0.10 |  | 0.02 | 0.791 | -0.11, 0.14 |  | 0.00 | 0.949 | -0.11, 0.12 |  |
|  | (0.06) |  |  |  | (0.06) |  |  |  | (0.06) |  |  |  |
| Origin Beliefs |  |  |  |  | 0.57*** | 0.000 | 0.51, 0.63 |  | -0.19*** | 0.000 | -0.25, -0.13 |  |
|  |  |  |  |  | (0.03) |  |  |  | (0.03) |  |  |  |
| Constant (Control) | 4.49*** | 0.000 | 4.11, 4.88 |  | 3.05*** | 0.000 | 2.56, 3.53 |  | 5.50*** | 0.000 | 5.06, 5.95 |  |
|  | (0.20) |  |  |  | (0.25) |  |  |  | (0.23) |  |  |  |
| N | 1071 |  |  |  | 1071 |  |  |  | 1071 |  |  |  |
| *AIC* | 3806.5 |  |  |  | 3877.9 |  |  |  | 3711.9 |  |  |  |
| *BIC* | 3846.3 |  |  |  | 3922.7 |  |  |  | 3756.6 |  |  |  |

*Note:* Cell entries are OLS coefficients with standard errors in parentheses below. Two-tailed *p*-values and confidence intervals are presented in the adjacent columns.

** p<0.10, ** p<0.05, *** p<0.01*

**Appendix: Alternative Model Specification – Treatment Effects and Partisanship**

***Table A5: Personal Steps – Condition Assignment and Party Identification***

|  | *Coef.* | *p-value* | *95% CI* |
| --- | --- | --- | --- |
| Natural Origin | -0.47** | 0.048 | -0.93, -0.00 |
|  | (0.24) |  |  |
| Chinese Conspiracy | -0.51** | 0.027 | -0.96, -0.06 |
|  | (0.23) |  |  |
| Competitive Frame | -0.47** | 0.037 | -0.92, -0.03 |
|  | (0.23) |  |  |
| Party Identification | 0.10*** | 0.005 | 0.03, 0.17 |
|  | (0.03) |  |  |
| Natural Origin X Party Identification | 0.06 | 0.211 | -0.04, 0.16 |
|  | (0.05) |  |  |
| Chinese Conspiracy X Party Identification | 0.06 | 0.237 | -0.04, 0.16 |
|  | (0.05) |  |  |
| Competitive Frame X Party Identification | 0.06 | 0.228 | -0.04, 0.16 |
|  | (0.05) |  |  |
| Constant (Control) | 5.71*** | 0.000 | 5.40, 6.02 |
|  | (0.16) |  |  |
| N | 1071 |  |  |
| *AIC* | 3351.7 |  |  |
| *BIC* | 3391.5 |  |  |

*Note:* Cell entries are OLS coefficients with standard errors in parentheses below. Two-tailed *p*-values and confidence intervals are presented in the adjacent columns.

** p<0.10, ** p<0.05, *** p<0.01*
